# Noncontact Acoustic Vibration Method for Firmness Evaluation in Multiple Peach Cultivars

**DOI:** 10.3390/foods14223899

**Published:** 2025-11-14

**Authors:** Dachen Wang, Laili Li, Tao Shi, Jun Cao, Xuesong Jiang, Hongzhe Jiang, Zhe Feng, Hongping Zhou

**Affiliations:** 1 Jiangsu Co-Innovation Center of Efficient Processing and Utilization of Forest Resources, Nanjing Forestry University, Nanjing 210037, China; 2 Key Laboratory of on Site Processing Equipment for Agricultural Products, Ministry of Agriculture, Hangzhou 310058, China; 3 College of Mechanical and Electronic Engineering, Nanjing Forestry University, Nanjing 210037, China; 4 School of Electrical Engineering and Automation, Hubei Normal University, Huangshi 435005, China

**Keywords:** nondestructive assessment, peach firmness, acoustic vibration, multi-cultivar models, one-dimensional convolutional neural network

## Abstract

Peach firmness is a critical quality attribute, yet conventional destructive measurement methods are unsuitable for batch detection in industrial settings. This study investigated a noncontact method for firmness assessment across multiple peach cultivars based on acoustic vibration technology. Three peach cultivars were mechanically excited via a controlled air jet, and the resulting acoustic vibration responses were captured noninvasively using a laser Doppler vibrometer. The frequency-domain acoustic vibration spectra were used as input for firmness prediction models developed using partial least squares regression (PLSR), support vector regression (SVR), and a one-dimensional convolutional neural network (ISNet-1D) that incorporated Inception and squeeze-and-excitation modules. Comparative analysis demonstrated that the ISNet-1D substantially outperformed the conventional linear and nonlinear methods on an independent test set, achieving superior predictive accuracy, with a coefficient of determination ( $R_{P}^{2})$ of 0.8069, a root mean square error (RMSEP) of 0.9206 N/mm, and a residual prediction deviation ( ${RPD}_{P})$ of 2.2879. The good performance of the ISNet-1D can be attributed to the integration of multi-scale convolutional filters with a channel-wise attention mechanism. This integration allows the network to adaptively prioritize discriminative spectral features, thereby enhancing its prediction accuracy. A hierarchical transfer learning strategy was proposed to improve model generalizability, offering a practical and cost-effective means to adapt to diverse cultivars. In summary, the combination of noncontact acoustic vibration and deep learning presents a robust, accurate, and nondestructive methodology for assessing peach firmness, demonstrating considerable potential for cross-cultivar application in industrial sorting and quality control.

## 1. Introduction

Peach (*Prunus persica* L.) is valued for its distinctive flavor and nutritional richness [1]. It contains vitamins, dietary fiber, minerals, and bioactive compounds, which contributes to its widespread consumption worldwide [2]. As consumer expectations for fruit quality continue to rise, the market demand for high-quality peaches has increased. However, the peach industry still faces challenges of inconsistent fruit quality and imbalances between supply and demand, which underscores the importance of efficient quality control. Fruit quality is commonly evaluated based on appearance, flavor, texture, and nutritional value [3]. Appearance encompasses visual traits such as size, shape, color, and surface integrity. Flavor, a more complex attribute, results from the interplay of sweetness, acidity, bitterness, astringency, and aromatic compounds. Texture refers to the mechanical and structural properties perceived through tactile and oral sensations. Among these quality indicators, firmness is a key texture attribute used to evaluate peach maturity, sensory quality, and postharvest shelf life [4]. Therefore, the development of rapid, nondestructive techniques for measuring firmness is essential for accurate fruit grading and supply chain optimization.

The acoustic vibration response of fruit is closely related to its internal physical properties [5], providing a viable approach for firmness assessment. In mechanical terms, fruit can be modeled as a vibration system consisting of mass and elastic components [6]. By analyzing dynamic response characteristics, such as resonance frequency and vibration attenuation under external excitation, key mechanical properties of the system can be derived. Abbott et al. [7] pioneered the use of acoustic vibration technology for evaluating apple firmness and introduced the elasticity index *EI* = *f*_2_^2^*m*, where *f*_2_ is the second resonance frequency and *m* is the mass of the sample. Since then, acoustic vibration technology has been widely applied to assess various fruit quality attributes, including firmness [8], internal decay [9], and split-pit defects [10].

A typical acoustic vibration detection system comprises an excitation unit, a signal acquisition unit, and a signal processing module. Commonly used excitation methods include tapping [11], electromagnetic shaker [12], loudspeaker [13], and laser-induced plasma (LIP) [14]. Among these, tapping is simple to perform but may cause mechanical damage; an electromagnetic shaker offers stable output but lacks portability; loudspeaker and LIP enable noncontact excitation but are limited by insufficient excitation intensity or sensitivity to environmental interference. In 2024, a noncontact excitation method based on an air jet was proposed [15], which was suitable for in-field vibration testing of peaches on the tree. This method features a short action time, a controllable impact force, and non-destructiveness, demonstrating considerable potential for indoor applications.

The acquisition of acoustic vibration signals primarily relies on contact sensors (e.g., accelerometers, piezoelectric sensors) and noncontact sensors (e.g., microphones, laser Doppler vibrometers) [16]. Although contact sensors are commonly used in practice, their added mass may alter the fruit’s natural vibration behavior. Zhang et al. [17] developed a wearable acoustic device for predicting kiwifruit firmness using the stiffness index, which showed a good correlation with sensory firmness. In contrast, noncontact methods are more suitable for high-throughput detection. Kataoka et al. [18] assessed tomato firmness using a speaker–microphone system, but this approach was susceptible to ambient noise. The laser Doppler vibrometer (LDV), known for its high resolution and wide dynamic range [19], enables precise measurement of fruit surface vibrations. Furthermore, with recent advances in artificial intelligence, deep learning models have shown considerable potential in feature extraction and modeling of acoustic vibration signals and have been successfully applied to predicting the postharvest quality of fruits [20,21]. However, the generalization capability and prediction accuracy of deep learning models are often limited when applied across different cultivars or growing conditions due to biological variability and signal inconsistency. Most existing models are developed for specific cultivars and lack generalizability, which limits their broad adoption in the fruit industry.

To achieve rapid, nondestructive, and accurate firmness evaluation across multiple peach cultivars, this study proposes a noncontact acoustic vibration method with deep learning algorithms to construct a cross-cultivar firmness prediction model. The specific objectives are (1) to develop a noncontact acoustic vibration signal acquisition system that combines an air jet and an LDV; (2) to analyze commonalities and differences in acoustic vibration characteristics across different peach cultivars; (3) to propose a deep learning framework for cross-cultivar firmness assessment based on acoustic vibration spectra; and (4) to explore a transfer learning-based strategy for model updating, providing a practical pathway to enhance generalizability across new cultivars.

## 2. Materials and Methods

### 2.1. Plant Materials

**Figure 1:**
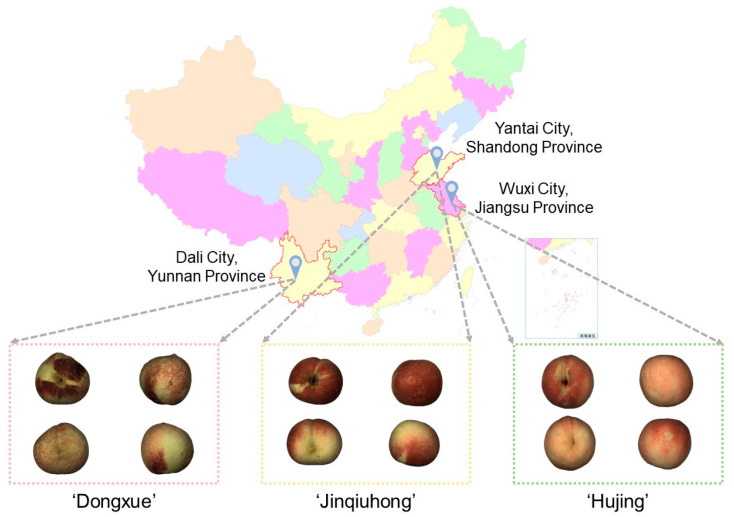
Geographic origins of the three peach cultivars.

The experimental materials consisted of three peach (*Prunus persica* (L.) Batsch) cultivars (Figure 1). In September 2024, a total of 128 ‘Hujing’ peaches were obtained from Wuxi, Jiangsu Province, China. Subsequently, 167 ‘Jinqiuhong’ peaches were collected from Longkou, Yantai, Shandong Province, China, in November 2024. Then, 176 ‘Dongxue’ peaches were collected from Dali, Yunnan Province, China, in December 2024. All samples underwent a standardized postharvest procedure involving immediate storage at 4 °C. Before vibration data collection, the surface of each fruit was rinsed with tap water and dried with paper towels to reduce interference from surface trichomes. The samples were removed from 4 °C storage and allowed to equilibrate for one hour under controlled laboratory conditions at 20 ± 2 °C and 65 ± 5% relative humidity. A combined dataset of 471 peaches from the three cultivars was thus established. The measurements for Hujing peaches were conducted on days 1, 3, 5, and 7 after transportation to the laboratory. For Jinqiuhong and Dongxue peaches, the measurements were performed on days 1, 3, 5, 7, and 9 after transportation to the laboratory. The firmness and acoustic vibration data of all samples were collected and are described below.

### 2.2. Acoustic Vibration Data Acquisition

**Figure 2:**
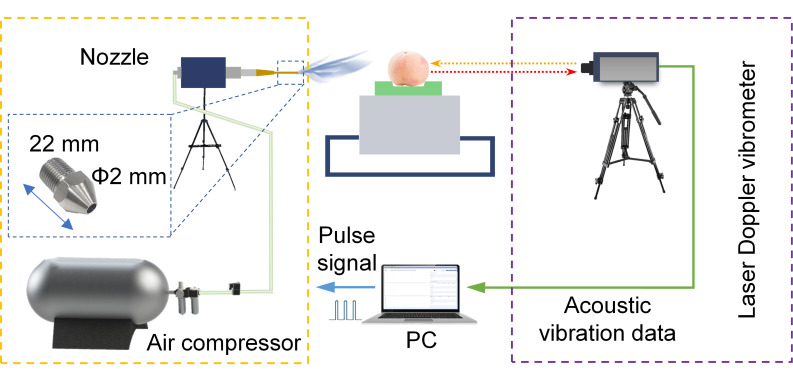
Schematic illustration of a noncontact acoustic vibration detection system.

As illustrated in Figure 2, the noncontact acoustic vibration system consisted of an air-jet excitation module and a signal acquisition module. The air-jet excitation module comprised an air nozzle, an air compressor (E8L, Zhejiang Jonway Machinery & Electric Manufacture Co., Ltd., Taizhou, China), a solenoid valve (2V025, Delixi Group Co., Ltd., Yueqing, China), and a pressure-reducing valve (QTY08, Delixi Group Co., Ltd., Yueqing, China). This module was designed to deliver high-pressure air pulses to mechanically excite the peach. The acquisition module included an LDV (MV-HW-TRLC, Zhigan Photon Technology Co., Ltd., Suzhou, China) and a personal computer. Data acquisition was conducted using the proprietary software of the LDV, which enabled configuration of sampling parameters and recording of acoustic vibration signals. During testing, each peach underwent three excitations at 5 s intervals, and the acoustic vibration data were captured simultaneously. The excitation module and the acquisition module were positioned on opposite sides of the equatorial plane to enhance efficiency and obtain consistent signals with high signal-to-noise ratio. The excitation pressure and duration were set to 300 kPa and 200 ms, respectively, with the nozzle located 6 cm from the fruit surface. The sampling frequency was fixed at 312.5 kHz.

**Figure 3:**
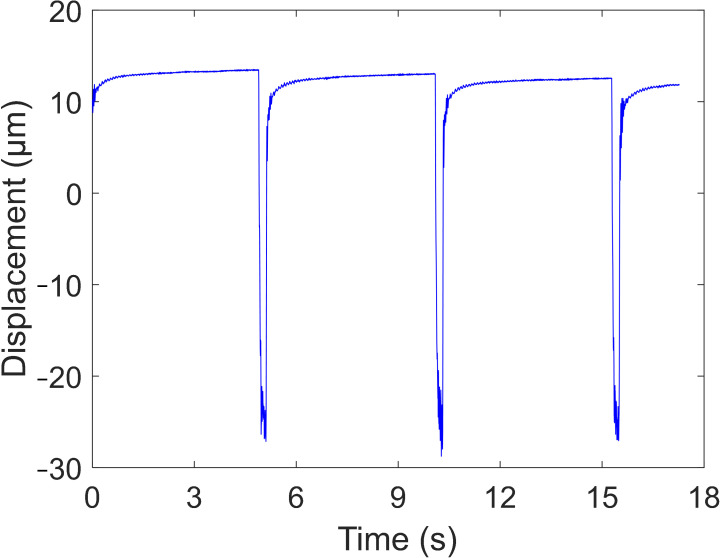
A typical time-domain signal of a peach measured by the noncontact acoustic vibration detection system.

Figure 3 displays a representative time-domain acoustic vibration signal acquired from a peach sample. Upon activation of the air-jet excitation module, the peach began to vibrate, with the vibration amplitude rapidly increasing to a peak before gradually decaying. To reduce computational load while retaining essential vibrational information, signal segments spanning from 1.5 s before to 1.5 s after the onset of excitation were extracted from the complete time-series data. The extracted signals were subsequently analyzed using an autoregressive (AR) model combined with the Burg algorithm [22] to estimate the power spectral density (PSD). The PSD describes the distribution of signal power per unit frequency band and can be estimated without prior knowledge of the input excitation. The order of the AR model was set to 400 based on the smoothness of the PSD curve and the distinctness of spectral peaks. Consequently, a PSD comprising 2623 data points over a frequency range of 0–5000 Hz was obtained for each peach.

### 2.3. Reference Measurements of Firmness

**Figure 4:**
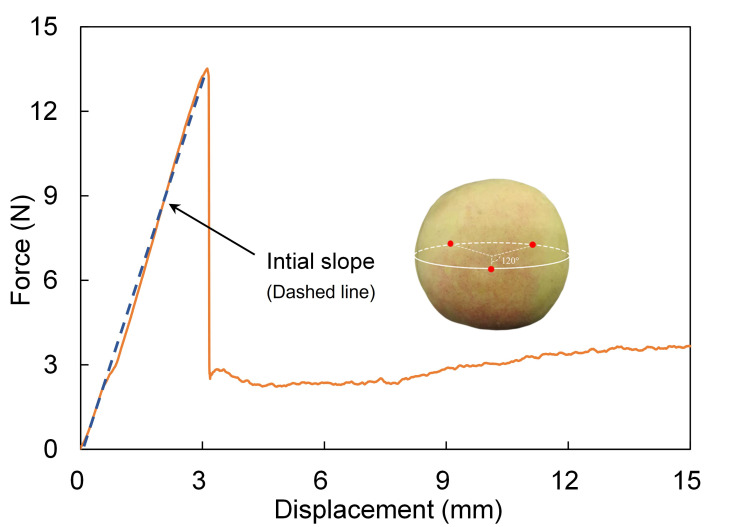
Reference peach firmness value derived from the force-displacement curve.

The reference values of peach firmness were determined using a texture analyzer (TA-XT2i, Stable Micro Systems Ltd., Godalming, UK) equipped with a 2 mm diameter cylindrical probe. Puncture tests were conducted at three points spaced 120° apart along the equatorial region of each fruit. Under a loading speed of 1 mm/s and a penetration distance of 15 mm, force-displacement curves were obtained for each test location. The initial slope of each force-displacement curve was calculated, and the average value from the three measurement points was taken as the reference firmness value of the peach (Figure 4). The initial slope was a measure of rigidity and was fundamentally related to the tissue’s structural integrity and cell wall strength [23]. The puncture tests were conducted immediately after the acoustic vibration measurements to prevent significant changes in fruit firmness between the two assessments. Ultimately, a total of 471 sets of experimental acoustic vibration data and corresponding firmness values were acquired. The dataset was divided into training, validation, and test sets in a ratio of 7:1:2 [24]. Since the validation set was used for relative comparison, a 10% allocation was adequate for guiding hyperparameter tuning without significantly reducing the training set size or impairing the model’s learning capacity.

### 2.4. Modeling Approaches for Peach Firmness Prediction

#### 2.4.1. Partial Least Squares Regression (PLSR)

A partial least squares regression (PLSR) model was used as a baseline linear method to assess the linear relationship between acoustic vibration features and peach firmness. PLSR is a classical method for handling high-dimensional, collinear spectral-type data [25]. The raw acoustic vibration spectra were first standardized to eliminate scale effects. Subsequently, all 2623 data points from the full spectrum were directly used as independent variables for the model. PLSR operated by projecting the predictors and response into a new set of latent variables (LVs) that maximized the covariance between the vibration signals and firmness values. The optimal number of LVs was determined by minimizing the RMSE of the validation set to prevent overfitting and ensure model robustness. The PLSR method provided an interpretable, linear baseline. Its limited performance suggested the presence of more complex, nonlinear characteristics within the data.

#### 2.4.2. Support Vector Regression (SVR)

Based on the structural risk minimization principle, the support vector regression (SVR) model exhibits good generalization capability [26]. An SVR model was employed to characterize the potential nonlinear relationship between acoustic vibration features and peach firmness. Due to the high dimensionality of the input features, principal component analysis was performed for dimensionality reduction. Principal components that collectively accounted for more than 95% of the total variance were retained as model inputs, which helped mitigate the curse of dimensionality and enhanced computational efficiency. The choice of kernel function was crucial in determining the performance and generalization capability of the SVR model. Common kernels include the linear kernel for linear problems, the polynomial kernel for feature interactions, the Gaussian kernel for local patterns, and the sigmoid kernel as a neural network analog. Hyperparameters, including kernel function, box constraint, and epsilon (ε), were optimized through a Bayesian optimization algorithm with the validation set.

#### 2.4.3. One-Dimensional Inception-Squeeze-and-Excitation Network (ISNet-1D)

**Figure 5:**
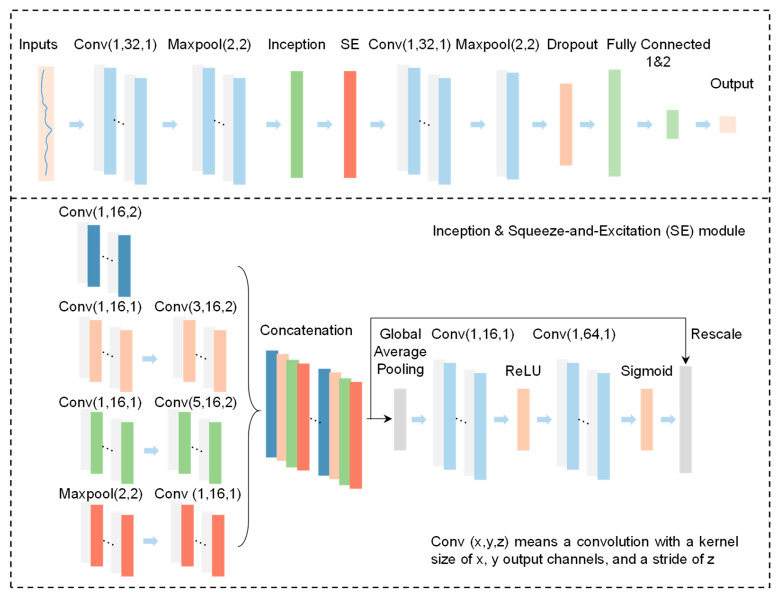
Architecture of the proposed ISNet-1D for peach firmness prediction.

To investigate advanced methods for predicting peach firmness, a one-dimensional convolutional neural network (ISNet-1D) incorporating an Inception module and a squeeze-and-excitation (SE) module was developed [27,28]. As shown in Figure 5, the network received one-dimensional PSDs of length 2623 through its input layer. An initial convolutional layer consisting of 16 filters with a kernel size of 3 was applied to extract preliminary features from the input spectra. Subsequent processing included batch normalization, a ReLU activation function, and a max-pooling layer to condense the feature representation. The core architecture employed a multi-branch Inception structure for parallel feature extraction. The input features were processed through four distinct pathways: (1) a single convolution with a kernel size of 1 for linear projection and channel reduction; (2) a sequential convolution with a kernel size of 1 followed by a second convolution with a kernel size of 3, capturing medium-range correlations; (3) a convolution with a kernel size of 1 followed by another with a kernel size of 5, integrating broader contextual information; and (4) a max-pooling layer followed by a convolution with a kernel size of 1 to preserve original features while adjusting dimensionality. The outputs of all four pathways were subsequently depth-concatenated, synthesizing a multi-scale feature map that incorporated vibrational patterns extracted across varying receptive fields.

Following the Inception module, an SE attention block was introduced to model channel-wise dependencies. Within this block, a ‘squeeze’ operation was first performed via global average pooling to produce channel-wise statistics. Then, an ‘excitation’ operation was implemented by two successive convolutions with a kernel size of 1, resulting in a set of adaptive weights. These weights recalibrated the original feature maps, enabling the network to selectively emphasize informative channels relevant to firmness. The refined features were subsequently processed by a convolutional layer with 32 filters (kernel size 5) and a max-pooling layer for further abstraction. The network culminated in a fully connected layer with 64 units, which incorporated dropout regularization (rate = 0.3) to mitigate overfitting. A final regression layer outputted a continuous value representing the predicted firmness. By integrating multi-scale feature extraction with channel-wise attention, the proposed architecture was designed to effectively model the complex relationships between vibrational signatures and the mechanical properties of peaches.

The model was trained using the adaptive moment estimation (Adam) optimizer, with the specific hyperparameters listed in Table 1. To enhance generalization and mitigate overfitting, an early stopping criterion was employed, and the model weights from the epoch with the lowest validation loss were retained for final evaluation.

The model was trained by minimizing a loss function comprising a mean squared error and an L2 regularization terms (Equation (1)). The composite loss function quantifies the discrepancy between the predicted and true firmness values while penalizing large weight magnitudes to mitigate overfitting.

(1) $$Loss=\frac{1}{n}\sum_{i=1}^{n}{{(y}_{i}-\hat{y_{i}})}^{2}+\frac{1}{2}\lambda ∥\omega {∥}^{2}$$

where $y_{i}$ represents the reference firmness value of the *i*-th sample, $\hat{y_{i}}$ denotes the corresponding predicted firmness value, and *n* indicates the total number of samples in the dataset. The variable **ω** corresponds to the weight vector of the model, while *λ* is the regularization coefficient that controls the penalty strength applied to the magnitude of the weights to mitigate overfitting.

### 2.5. Model Evaluation

The performance of the peach firmness regression models was evaluated using the coefficient of determination (*R*^2^), the root mean squared error (RMSE), and the residual prediction deviation (*RPD*) [29,30]. *R*^2^ represents the proportion of variance in the observed data explained by the model, with values closer to 1 indicating a better fit. RMSE measures the average prediction error in the original units of the response variable, where lower values reflect higher accuracy. *RPD*, defined as the standard deviation of the reference data divided by the RMSE, relates the model error to the natural variability in the dataset; higher *RPD* values indicate stronger predictive ability and practical robustness. The calculation equations for the three metrics are as follows:

(2) $$RMSE=\sqrt{\frac{\sum_{i=1}^{n}{{(y}_{i}-\hat{y_{i}})}^{2}}{n}\;}$$

(3) $$R^{2}=1-\frac{\sum_{i=1}^{n}{{(y}_{i}-\hat{y_{i}})}^{2}\;}{{\sum_{i=1}^{n}{(y}_{i}-\bar{y})}^{2}}$$

(4) $$RPD=\frac{SD}{RMSE}$$

$y_{i}$ is the measured firmness value, $\hat{y_{i}}$ is the predicted firmness value, $\bar{y}$ is the mean of the measured values, *n* is the number of samples, and *SD* is the standard deviation of the measured firmness values.

## 3. Results

### 3.1. Statistical Analysis of Acoustic Vibration Spectra and Peach Firmness

**Figure 6:**
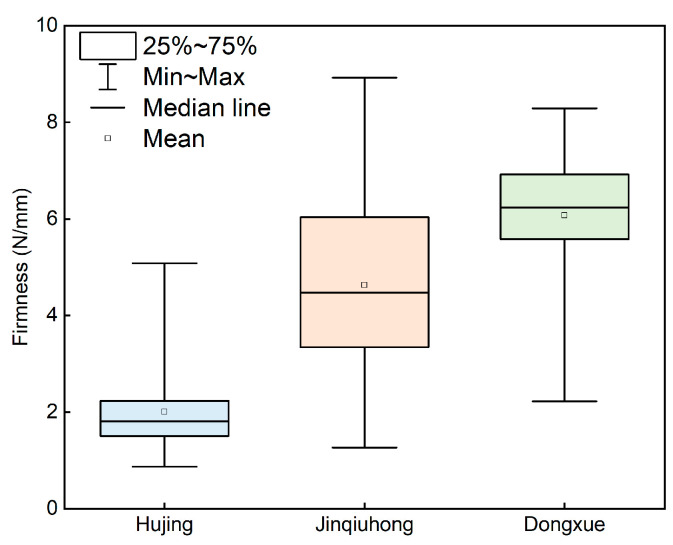
Statistical results of reference firmness values for three peach cultivars.

The statistical results of reference firmness values for three peach cultivars are shown in Figure 6. The data revealed a discernible gradient in firmness among the cultivars. Hujing exhibited the lowest firmness with a mean value of 2.01 N/mm, and its values were predominantly clustered within the lower range of 0.87–5.08 N/mm. Jinqiuhong displayed intermediate firmness with a mean value of 4.63 N/mm and exhibited the widest distribution range among the three cultivars (1.26–8.92 N/mm). In contrast, Dongxue demonstrated the highest firmness with a mean value of 6.07 N/mm, and its lower limit was notably higher than that of the other cultivars (2.22–8.29 N/mm).

**Figure 7:**
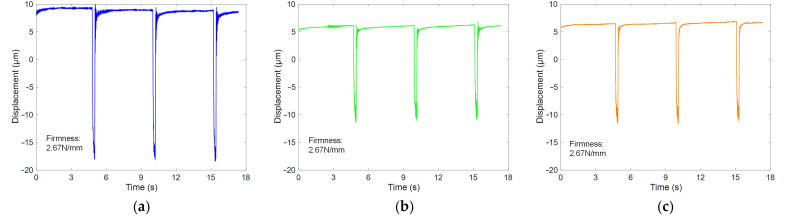
Time-domain vibration response signals and corresponding firmness values of Hujing (**a**), Jinqiuhong (**b**), and Dongxue (**c**) peaches.

Figure 7 displays representative time-domain response signals and their corresponding firmness values for the ‘Hujing’, ‘Jinqiuhong’, and ‘Dongxue’ peach cultivars, as measured by the developed system. The results indicated that the system effectively induced vibrational responses in peaches across three cultivars. However, comparative analysis revealed significant differences in vibration peak amplitudes among the cultivars, even when their firmness values were similar. These findings demonstrated that time-domain features, such as signal amplitude, could not reliably characterize fruit firmness. Therefore, to better extract firmness-related features of the fruit, subsequent analysis was shifted to the frequency domain.

**Figure 8:**
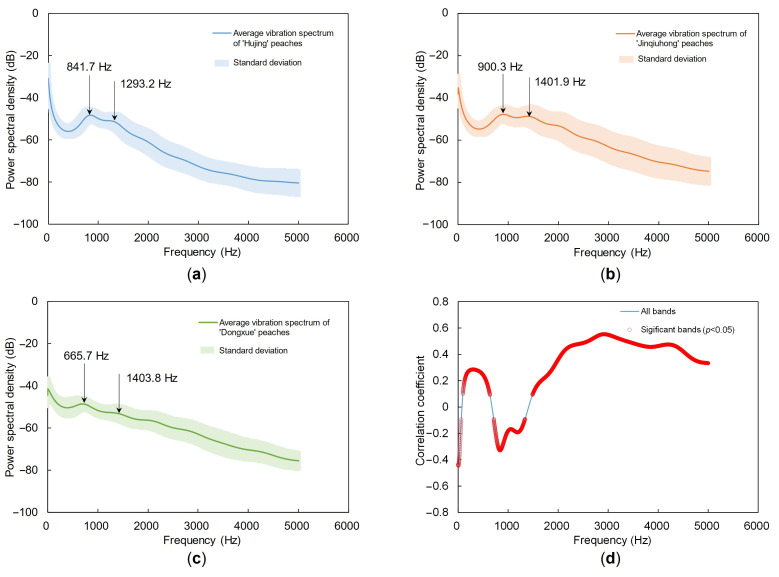
Average acoustic vibration spectra of Hujing (**a**), Jinqiuhong (**b**), and Dongxue (**c**) peaches. (**d**) Correlation analysis between the full acoustic vibration spectra and firmness across all samples.

The average PSDs of Hujing, Jinqiuhong, and Dongxue peaches are presented in Figure 8. The first and second resonant frequencies were identified as 841.7 Hz and 1293.2 Hz for Hujing, 900.3 Hz and 1401.9 Hz for Jinqiuhong, and 665.7 Hz and 1403.8 Hz for Dongxue, respectively. Notably, Dongxue showed the lowest first resonant frequency despite having the highest firmness, deviating from the generally anticipated positive correlation between firmness and resonant frequency. The results indicated that the relationship between firmness and resonant frequency was not straightforward and can be cultivar-dependent. This discrepancy may be attributed to the complex internal structure and anisotropic mechanical properties of peaches. Resonant frequency is influenced not only by firmness but also by multiple factors, including cultivar, size, shape, density, and internal architecture. The complex interactions among these factors may lead to a non-monotonic relationship between firmness and resonant frequency across different cultivars.

A correlation analysis was conducted between the full PSDs and firmness across all samples. As shown in Figure 8d, the correlation exhibited clear frequency-dependent behavior. In the 0–70 Hz and 700–1300 Hz ranges, correlation coefficients were generally negative, indicating that vibration amplitude decreased with increasing firmness. In contrast, above 1500 Hz, the correlation coefficients became positive and increased with frequency, reaching approximately 0.55 near 3000 Hz. These patterns suggested that low-frequency responses were more affected by mass and damping properties, resulting in a negative correlation with firmness, whereas high-frequency responses were more sensitive to tissue elasticity, leading to a positive correlation. The frequency band near 3000 Hz, which showed the highest correlation with firmness, did not coincide with the resonant peaks of the three cultivars. Therefore, prediction models relying solely on resonant frequencies or specific parameters may have limited generalizability when applied across cultivars with distinct physical properties. In conclusion, utilizing full PSDs as model inputs supports the development of more robust and transferable firmness prediction models that are less dependent on cultivar-specific characteristics.

### 3.2. Multi-Cultivar PLSR and SVR Models for Peach Firmness Prediction

Table 2 summarizes the prediction results of peach firmness using PLSR, SVR, and ISNet-1D models with full PSDs as inputs. Overall, the SVR model exhibited superior predictive performance and robustness over the PLSR model. During modeling, the optimal number of latent variables for the PLSR model was set to 49 by minimizing the RMSE of the validation set. The key hyperparameters of the SVR model were obtained using the Bayesian optimizer, including a linear kernel, a box constraint of 0.0109, and an epsilon value of 0.0086.

On the calibration set, the SVR model achieved a higher $R_{C}^{2}$ of 0.7111 and a lower RMSEC of 1.1260 N/mm than the PLSR model ( $R_{C}^{2}$ = 0.6665, RMSEC = 1.1812 N/mm), indicating better fitting performance. Moreover, the *RPD_C_* of the SVR model (1.8706) also exceeded that of the PLSR model (1.7338), reflecting greater model stability. For the test set, the $R_{P}^{2}$, RMSEP, and ${RPD}_{P}$ of the SVR model were 0.6827, 1.1277 N/mm, and 1.8161, respectively. The $R_{P}^{2}$, RMSEP, and ${RPD}_{P}$ of the PLSR model were 0.6546, 1.2312 N/mm, and 1.7107, respectively. In summary, using full PSDs as inputs, the SVR model surpassed PLSR in predicting peach firmness. However, as the *RPD* values of both models remained below 2.0, there was still potential for further enhancing predictive performance.

### 3.3. Multi-Cultivar ISNet-1D for Peach Firmness Prediction

To further enhance prediction accuracy, a 1D-CNN architecture incorporating an Inception module and a squeeze-and-excitation attention mechanism (ISNet-1D) was specifically designed to process raw PSDs. This hierarchical structure enabled the model to automatically learn and integrate features directly from the signal, spanning broad resonance patterns to fine-grained textural components. The proposed ISNet-1D demonstrated significantly improved performance compared to conventional machine learning approaches. On the test set, the ISNet-1D achieved significant improvements over the SVR model, with an 18.19% higher $R_{P}^{2}$ (0.8069), an 18.36% lower RMSEP (0.9206 N/mm), and a 25.98% higher ${RPD}_{P}$ (2.2879). Notably, an *RPD_P_* value exceeding 2.0 suggested good predictive capability and robustness for practical applications.

**Figure 9:**
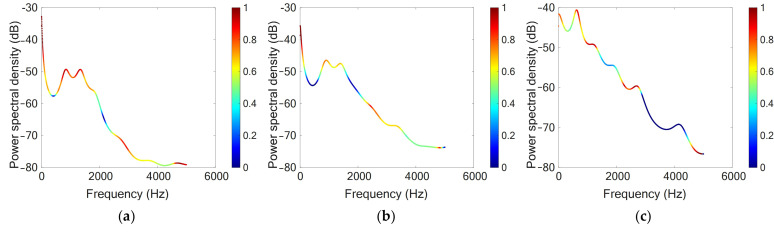
Visualization of typical peach power spectral density (PSD) for Hujing (**a**), Jinqiuhong (**b**), and Dongxue (**c**) peaches using the gradient-weighted class activation mapping method.

The gradient-weighted class activation mapping (Grad-CAM) method was applied to enhance the interpretability of the ISNet-1D [31]. It was used to generate visual heatmaps by weighting and upsampling the feature maps from the final convolutional layer. As shown in Figure 9, the relative importance of different regions in a typical PSD was represented by a color gradient from blue to red, with red indicating higher relevance. The results demonstrated that areas adjacent to the first two resonant frequencies were particularly important for predicting firmness, consistent with the physical principle that resonant frequency correlated with material stiffness. However, comparisons across three peach cultivars revealed that the relationship between resonant frequencies and firmness was neither strictly linear nor uniformly positive. Consistent with this, the heatmap suggested that not all resonant frequencies contributed equally to the model’s decisions.

The ISNet-1D also assigned importance to spectral troughs and specific non-resonant frequency bands (e.g., 0–280 Hz and 4600–5120 Hz), which were typically overlooked in conventional parameter-based methods. These findings suggested that the model leveraged complementary information across the entire spectrum to make its decisions, contributing to its superior performance. Unlike PLSR and SVR methods, the ISNet-1D learned discriminative features directly from raw acoustic vibration spectra. By integrating multi-scale convolutional filters with a channel-wise attention mechanism, the network adaptively prioritized both global resonant patterns and localized high-frequency components that were closely related to peach firmness. This end-to-end learning framework reduced potential information loss associated with manual feature engineering and captured complex, nonlinear relationships in the PSDs more effectively.

### 3.4. Exploration of Model Generalizability via Transfer Learning

To evaluate the generalizability of the original model, which was trained on data from three cultivars over a single growing season, an external validation was conducted using an independent set of 120 fruit samples of the ‘Baifeng’ peach cultivar. These samples were collected in July 2025 from an orchard in Wuxi, Jiangsu Province, following the same experimental procedures described in the Section 2. The firmness values for the external dataset ranged from 0.45 to 4.47 N/mm, with a mean of 1.79 N/mm and a standard deviation of 1.07 N/mm (Table 3). The initial predictions generated by the original ISNet-1D for this previously unseen cultivar exhibited limited accuracy, indicating cultivar-specific dependency and poor cross-cultivar transferability.

A hierarchical transfer learning strategy was implemented to update the model. After loading the pretrained ISNet-1D, the weights of all deep convolutional layers were frozen to preserve generic vibrational features learned from the original dataset. The last convolutional layer was made trainable with a reduced learning rate, allowing for adjustments to higher-level abstract features relevant to the new cultivar. Meanwhile, the two fully connected layers at the network’s output were set as fully trainable under standard learning rates to recalibrate the feature combination weights for better accommodation of inter-cultivar variability. The new dataset was split into training and validation subsets at a 4:1 ratio. Subsequently, the model was fine-tuned in an end-to-end manner via transfer learning and independently validated. After transfer learning, a significant improvement in model performance was observed. For the calibration set, the $R_{C}^{2}$, RMSEC, and *RPD_C_* were 0.7500, 0.5368 N/mm, and 2.2503, respectively. For the validation set, the $R_{V}^{2}$, RMSEV, and *RPD_V_* were 0.7120, 0.5370 N/mm, and 1.9304, respectively. The results indicated that the hierarchical transfer learning approach significantly mitigated the initial decline in predictive accuracy and improved the model’s adaptability to the new cultivar.

## 4. Discussion

Current acoustic vibration methods for fruit firmness evaluation predominantly rely on contact-based excitation or vibration measurement techniques and are generally applied to a single fruit cultivar. To achieve rapid and accurate firmness assessment across multiple peach cultivars, this study proposed a noncontact acoustic vibration detection system combining air-jet excitation and an LDV. Deep learning algorithms were used to develop a cross-cultivar model for accurate peach firmness prediction.

In terms of excitation approaches, sinusoidal sweep and impact excitation exhibit distinct characteristics. Sinusoidal sweep excitation delivers energy at one frequency at a time, effectively stimulating structural vibrations across all frequency points and aiding in the clear identification of even weak resonance peaks. However, this method is relatively time-consuming and not ideally suited for rapid detection. In contrast, impact excitation is faster and can deliver a broadband excitation. An ideal instantaneous impact exhibits a flat frequency spectrum over a theoretically infinite frequency range, distributing energy uniformly across an extremely broad band. Under practical conditions, impacts have finite duration, and the spectral width is inversely proportional to the pulse duration. Shorter pulses encompass richer high-frequency components, while longer pulses preserve more low-frequency energy but attenuate at higher frequencies. The instantaneous air jet employed in this work produces a very brief pulse. Although the energy per unit frequency is relatively low, experimental results indicated that this technique was capable of acquiring acoustic vibration spectra with clearly identifiable resonant peaks for peaches. Subsequent research may focus on incorporating impact excitation into online inspection systems to improve the intelligence and efficiency of detection.

Analysis of acoustic vibration spectra obtained from multiple peach cultivars indicated that the relationship between resonant frequency and firmness was neither strictly linear nor consistently positive. Therefore, prediction models that rely exclusively on resonant frequency or other conventional spectral parameters may exhibit limited applicability when assessing firmness across different cultivars with divergent physical properties. To enhance prediction accuracy, a one-dimensional convolutional neural network (ISNet-1D) was designed, incorporating a multi-scale Inception module and the squeeze-and-excitation attention mechanism for processing raw PSDs. This end-to-end learning framework adaptively emphasized global resonance patterns and localized high-frequency components that were closely associated with peach firmness. Compared with PLSR and SVR models, ISNet-1D demonstrated good firmness prediction performance for multi-cultivar peaches.

Furthermore, the method proposed in this study is not restricted to peaches and can be extended to other spherical or near-spherical fruits, such as apples and pears. By utilizing the air-jet excitation and LDV-based acoustic vibration measurement approach, acoustic vibration data for specific fruits can be obtained. Adoption of the proposed deep learning architecture could further allow the development of prediction models for estimating firmness or other mechanical quality attributes tailored to specific fruit types. Future efforts should focus on evaluating the adaptability and generalization capacity of the model across a broader range of fruit species, and on optimizing the system for fully automated, high-throughput industrial sorting operations.

## 5. Conclusions

This study developed a noncontact methodology for the accurate evaluation of peach firmness across multiple cultivars. The approach combined air-jet excitation with the LDV to acquire acoustic vibration signals, along with a dedicated deep learning architecture (ISNet-1D) for firmness prediction. Experimental results showed that transient air-jet impact provided effective broadband excitation, producing high-quality spectral data suitable for rapid inspection. In comparative analyses, the ISNet-1D outperformed conventional machine learning methods. On the test set, ISNet-1D achieved an $R_{P}^{2}$ of 0.8069, an RMSEP of 0.9206 N/mm, and an *RPD_P_* of 2.2879. These values were 18.19%, 18.36%, and 25.98% higher than those of the SVR model, respectively. By incorporating multi-scale feature extraction and channel attention mechanisms, the ISNet-1D autonomously learned discriminative features from raw spectral data, enabling it to effectively capture the complex, nonlinear relationships between vibration characteristics and firmness. In conclusion, the integration of noncontact acoustic vibration detection with deep learning offers a feasible framework for assessing the firmness of specific peach cultivars.

However, the development of a universal and robust model applicable across multiple cultivars and growing seasons remains challenging and typically requires extensive datasets. Traditional chemometric models often exhibit performance degradation when applied to different cultivars or harvest years, frequently necessitating labor-intensive remodeling. The present study demonstrates that a transfer learning-based adaptation strategy potentially addresses this limitation. By leveraging a limited number of target-specific samples, the proposed approach maintains predictive performance while reducing data requirements, offering a practical pathway toward enhancing model generalizability in real-world applications. Future work will focus on extending and optimizing the method to a wider range of fruit types.

## Figures and Tables

**Table 1:** Configuration of hyperparameters for model training.

Hyperparameters	Value
Batch size	16
Initial learning rate	0.0001
Learning rate drop factor	0.1
Learning rate drop period	50
L2 regularization	0.0001
Max epochs	300

**Table 2:** Prediction results of multi-cultivar peach firmness by PLSR, SVR, and ISNet-1D models based on the frequency-domain acoustic vibration spectra.

Models	Calibration Set			Test Set		
	$R_{C}^{2}$	RMSEC (N/mm)	$RPD_{C}$	$R_{P}^{2}$	RMSEP (N/mm)	$RPD_{P}$
PLSR	0.6665	1.1812	1.7338	0.6546	1.2312	1.7107
SVR	0.7111	1.1260	1.8706	0.6827	1.1277	1.8161
ISNet-1D	0.8178	0.8371	2.4465	0.8069	0.9206	2.2879

**Table 3:** Statistical results of reference firmness values for ‘Baifeng’ peach cultivar.

Sample Set	Number of Samples	Firmness (N/mm)		
		Range	Mean	SD
All samples	120	0.45–4.47	1.79	1.07
Training set	96	0.48–4.47	1.83	1.08
Testing set	24	0.45–3.65	1.64	1.02

## Data Availability

The original contributions presented in this study are included in the article. Further inquiries can be directed to the corresponding authors.
